# Supplementary material for: From sequence to enzyme mechanism using multi-label machine learning
Source: BMC Bioinformatics. 2014 May 19;15:150. doi: 10.1186/1471-2105-15-150 (PMC4229970; doi:10.1186/1471-2105-15-150)
Supplement: Additional file 2 — Java code of ml2db. Additional file ml2db_code.tar.gz contains the Java source code to run the multi-label machine learning experiments and save the results to database. The code’s Javadoc is included. [file 1471-2105-15-150-S2.zip › additional file 2/ml2db/ml2db/doc/main/ml2db/al/database/QueryTable.html]

QueryTable


JavaScript is disabled on your browser.


- Overview
- Package
- Class
- Use
- Tree
- Deprecated
- Index
- Help

- Prev Class
- Next Class

- Frames
- No Frames

- All Classes

- Summary:
- Nested |
- Field |
- Constr |
- Method

- Detail:
- Field |
- Constr |
- Method


main.ml2db.al.database

## Class QueryTable

- java.lang.Object
- - uk.ac.standrews.utils.main.database.Table
  - - main.ml2db.al.database.QueryTable

- ---

    

  ```
  public class QueryTable
  extends uk.ac.standrews.utils.main.database.Table
  ```

  The table containing the query instances > the instances selected for
  labelling

  Version:
  :   12 Jul 2011

  Author:
  :   Luna De Ferrari ldf(at)st-andrews.ac.uk

- - ### Field Summary

    Fields

    | Modifier and Type | Field and Description |
    | --- | --- |
    | `static java.lang.String` | `INSTANCE_STRING` |
    | `static java.lang.String` | `QUERY_TABLE_NAME` |

    - ### Fields inherited from class uk.ac.standrews.utils.main.database.Table

      `m_writer`
  - ### Constructor Summary

    Constructors

    Constructor and Description

    `QueryTable()`
  - ### Method Summary

    Methods

    | Modifier and Type | Method and Description |
    | --- | --- |
    | `uk.ac.standrews.utils.main.database.TableRow` | `createRow(int experimentId, int stepLabelled, java.lang.String instance, java.lang.String timestamp, double utilityMetric, java.lang.String instanceString, uk.ac.standrews.utils.main.database.TableManager tableManager)` |
    | `uk.ac.standrews.utils.main.database.TableRow` | `createRow(java.lang.Object[] values, uk.ac.standrews.utils.main.database.TableManager manager)` |
    | `static java.util.Vector<uk.ac.standrews.utils.main.database.TableColumn>` | `getColumnDefinitions()` |

    - ### Methods inherited from class uk.ac.standrews.utils.main.database.Table

      `getColumnNames, getColumns, getDbManager, getTableCreator, getTableName, getTableReader, getTableRow, getTableWriter, hasKey, initialise, initialise, name, saveRow`
    - ### Methods inherited from class java.lang.Object

      `equals, getClass, hashCode, notify, notifyAll, toString, wait, wait, wait`

- - ### Field Detail


    - #### QUERY\_TABLE\_NAME

      ```
      public static final java.lang.String QUERY_TABLE_NAME
      ```

      See Also:
      :   Constant Field Values


    - #### INSTANCE\_STRING

      ```
      public static final java.lang.String INSTANCE_STRING
      ```

      See Also:
      :   Constant Field Values
  - ### Constructor Detail


    - #### QueryTable

      ```
      public QueryTable()
      ```
  - ### Method Detail


    - #### createRow

      ```
      public uk.ac.standrews.utils.main.database.TableRow createRow(int experimentId,
                                                           int stepLabelled,
                                                           java.lang.String instance,
                                                           java.lang.String timestamp,
                                                           double utilityMetric,
                                                           java.lang.String instanceString,
                                                           uk.ac.standrews.utils.main.database.TableManager tableManager)
      ```


    - #### createRow

      ```
      public uk.ac.standrews.utils.main.database.TableRow createRow(java.lang.Object[] values,
                                                           uk.ac.standrews.utils.main.database.TableManager manager)
      ```


    - #### getColumnDefinitions

      ```
      public static java.util.Vector<uk.ac.standrews.utils.main.database.TableColumn> getColumnDefinitions()
      ```


- Overview
- Package
- Class
- Use
- Tree
- Deprecated
- Index
- Help

- Prev Class
- Next Class

- Frames
- No Frames

- All Classes

- Summary:
- Nested |
- Field |
- Constr |
- Method

- Detail:
- Field |
- Constr |
- Method
